# *Warburgia ugandensis* Leaf and Bark Extracts: An Alternative to Copper as Fungicide against Downy Mildew in Organic Viticulture?

**DOI:** 10.3390/plants10122765

**Published:** 2021-12-14

**Authors:** Christian Kraus, Rada Abou-Ammar, Andreas Schubert, Michael Fischer

**Affiliations:** 1 Julius Kühn-Institute, Federal Research Centre of Cultivated Plants, Plant Protection in Fruit Crops and Viticulture, 76833 Siebeldingen, Germany; michael.fischer@julius-kuehn.de; 2 Fraunhofer Institute for Cell Therapy and Immunology (IZI), 04103 Leipzig, Germany; rada.abou-ammar@izi.fraunhofer.de (R.A.-A.); andreas.schubert@izi.fraunhofer.de (A.S.)

**Keywords:** botanical, Downy Mildew, *Plasmopara viticola*, viticulture, *Warburgia ugandensis*, plant extract, plant protection

## Abstract

In organic viticulture, copper-based fungicides are commonly used to suppress Downy Mildew infection, caused by the oomycete *Plasmopara viticola*. However, the frequent and intensive use of such fungicides leads to accumulation of the heavy metal in soil and nearby waters with adverse effects on the ecosystem. Therefore, alternative, organic fungicides against Downy Mildew are urgently needed to reduce the copper load in vineyards. In this study, the use of *Warburgia ugandensis* Sprague (Family Canellacea) leaf and bark extracts as potential fungicides against Downy Mildew were evaluated. In vitro (microtiter) and in vivo (leaf discs, seedlings) tests were conducted, as well as field trials to determine the efficacy of the extracts against Downy Mildew. The results revealed an MIC_100_ of 500 µg/mL for the leaf extract and 5 µg/mL for the bark extract. Furthermore, experiments with leaf discs and seedlings demonstrated a strong protective effect of the extracts for up to 48 h under (semi-) controlled conditions. However, in field trials the efficacy of the extracts distinctly declined, regardless of the extracts’ origin and concentration.

## 1. Introduction

Downy Mildew (DM) of grapevine, caused by the oomycete *Plasmopara viticola*, is one of the most threatening and harmful diseases in viticulture. Ever since it was introduced to European vineyards in 1876, probably by shipping of American grape cuttings, this pathogen caused massive yield losses in vine-growing regions all over the world [1,2]. Therefore, protective measures were urgently needed in the second half of the 19th century to control this disease, and it was a coincidence that led to the first plant protection product (PPP) against DM, the Bordeaux mixture [3]. Copper cations (Cu^2+^), which were the actual active substance against *P. viticola* in the mixture, then served as a basis for PPP to combat DM all over the world, mainly as Cu oxychloride, Cu sulfate and Cu hydroxide [4]. Furthermore, even almost 150 years after its discovery and after the development of other, more effective PPPs against DM, copper-based fungicides are still widely used in vineyards, especially in organic viticulture [2,5,6].

Although Cu is of considerable importance for crop protection, due to its high efficacy and reduced resistance risk, it has become a growing public concern over the last decades [6]. The continuous and intensive application of Cu-based fungicides leads to an accumulation of the heavy metal in agricultural soils and nearby waters and as a result negatively affects the ecosystems [2,7,8,9,10]. In organic viticulture, this problem is even more severe since alternatives with comparable efficacies to copper are rare presently or even non-existing since the ban of phosphonates [11]. Therefore, more PPPs are strongly needed to extend the “toolbox” for protection against DM and with that, reducing the cooper input in the environment [12].

One possible source of PPPs for organic agriculture is extracts derived from plant parts (e.g., leaves, bark, roots, fruits) or whole plants, so-called botanicals [13,14,15,16,17,18,19]. The interest in botanicals, not only as pesticides in agriculture but also as pharmaceuticals, massively increased in the last decades mostly due to public concerns regarding off-target effects by synthetic pesticides/drugs [20,21,22,23]. Also in viticulture, several plant extracts were evaluated regarding their efficacy against DM, with promising results: *Salvia officinalis* [24], *Vitis vinifera* [25], *Juncus effusus* [26], *Larix decidua* [27], *Verbesina lanata* [28], *Magnolia officinalis* [29], *Yucca schidigera*, *Glycyrrhiza glabra* [30]. However, despite strong efforts, so far, no PPP based on plant extracts could fulfill all criteria for a highly effective fungicide against DM in viticulture.

*Warburgia ugandensis* Sprague (Family Canellaceae; WU), commonly known as “Uganda Green Heart Tree”, is an evergreen plant, which is mainly distributed in East and South Africa [31,32,33]. For generations, traditional healers were using WU extracts made of bark, roots or leaves to treat different kinds of diseases/ailments like malaria, tuberculosis, skin diseases, ulcers, lung problems or intestinal worms, to name a few [31,34,35,36,37]. The reason for its high and diverse therapeutic potential is linked to a broad arsenal of phytochemicals belonging to the groups of drimane sesquiterpenes, tannins and mannitol [33,38,39]. Three of these phytochemicals are polygodial, warburganal and muzigadial, all showing antifungal activity against *Saccharomyces cerevisiae*, *Candida albicans*, *C. utilis*, and *Sclerotinia libertiana* [40,41]. Also, against the soil pathogens *Fusarium oxysporum*, *Alternaria passiflorae*, and *Aspergillus niger*, WU extracts showed inhibitory effects [42]. Besides antifungal properties, extracts made of WU exhibit further activities, e.g., antibacterial, antimycobacterial and antiplasmodial activities [31,34,43,44,45,46]. Concerning toxicity, a study conducted on mice classified WU extracts as non-cytotoxic and showed a lethal dose of (LD_50_) > 5000 mg/kg body weight [47]. Although highly concentrated WU extract was toxic to VERO cells, no toxicity was found by mammalian macrophage cells [35,48,49]. As a conclusion, extracts made of WU may be used as powerful active compounds in future PPPs against a wide variety of pathogens with minimal health risks. These properties are ideal requirements for an application of the extracts as fungicide in organic agriculture.

It was the aim of the present study to evaluate selected WU leaf (WLD) and bark (WBD) extracts as possible fungicide compounds against DM under in vitro and in vivo-conditions. Field trials were performed to compare the efficacy of WU extracts with a commercial Cu-based PPP. The obtained results will show whether WU extracts are adequate alternatives and thus can help to decrease the cooper load in organic viticulture.

## 2. Results

### 2.1. Microtiter Assay

**Figure 1:**
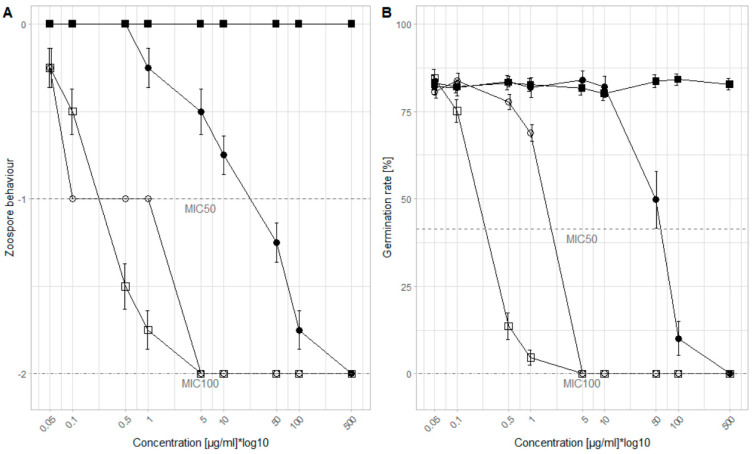
Results of the microtiter assays, (**A**) zoospore behavior and (**B**) germination rate of sporangia (*n* = 16): Control (■), Cu-fungicide (□), WLD (●), WBD (○). Three behavior categories were defined to measure the zoospore behavior: (0) normal zoospore motion; (−1) no or unusual zoospore motion; (−2) no zoospore release. Nine concentrations of each extract were selected to examine the inhibition efficacy: 0.05; 0.1; 0.5; 1; 5; 10; 50; 100; and 500 µg/mL. Y-axis is log10 transformed to generate a more coherent plot. MIC_50_ is indicated as a dashed line, MIC_100_ as a dotted-dashed line.

We tested the concentration range in which the WU extracts show an inhibitory effect against *P. viticola*. The MIC_50_ and MIC_100_ values for each extract were determined related to the zoospore behavior and the germination rate of the sporangia (Figure 1). Regarding the zoospore behavior, for the WLD, no germination (MIC_100_) appeared at 500 µg/mL. However, the zoospore mobility was still negatively affected (MIC_50_) at 25 µg/mL. The bark extract was more effective than the leaf extract with an MIC_100_ of 5 µg/mL and an MIC_50_ of 0.1 µg/mL. Compared to the Cu-fungicide, with an MIC_100_ of 5 µg/mL and an MIC_50_ of 0.25 µg/mL, the WBD had the same efficacy. Also in relation to the germination rate, the Cu-fungicide and the WBD share the same MIC_100_ of 5 µg/mL. However, the MIC_50_ of the Cu-fungicide was lower (0.25 µg/mL) compared to the WBD (2.5 µg/mL). For WLD, the efficacy was reduced when compared to the other two test products: at a concentration of 500 µg/mL all sporangia stopped germinating (MIC_100_) and at a concentration of 50 µg/mL the germination rate could nearly be halved (MIC_50_).

### 2.2. Leaf Disc Assay

**Figure 2:**
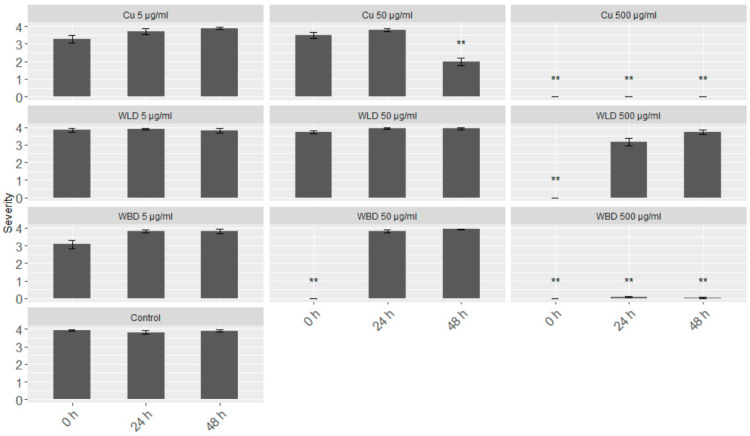
Merged (cv. ‘Müller–Thurgau’ and ‘Pinot Noir’) results of the leaf disc assays based on infection severity: (0) no sporulation, (1) minimal sporulation, (2) low sporulation, (3) medium sporulation, (4) full sporulation. For each treatment, three concentrations were tested (5, 50 and 500 µg/mL) and three different time points of sporangia inoculation (0, 24, and 48 h after spraying). Treatments were Cu-fungicide (Cu), WLD, WBD and control. Welch’s anova and Games–Howell test for multiple comparison: Significant differences of the treatments compared to control; ** *p* < 0.001; *n* = 48.

A leaf disc assay was chosen to test the efficacy of the WU extracts in an in vivo system. With three tested concentrations (5, 50 and 500 µg/mL) and three different infection time points (0, 24 and 48 h after treatment), the WLD had a protective effect only at the highest concentration, i.e., 500 µg/mL, and only if the inoculation took place directly after the treatment (0 h; Figure 2). When leaf discs were inoculated after 24 or 48 h, respectively, the leaf discs showed full sporulation (infection severity 4). WBD treatment resulted in a full protection or no sporulation (infection severity 0), respectively, when using 50 and 500 µg/mL, at 0 h. However, long-term protection, i.e., 24 and 48 h, only could obtained with 500 µg/mL WBD. Also, the Cu-fungicide at 500 µg/mL could maintain its protective effect up to 48 h. With 5 µg/mL, an infection could not be impeded, but with 50 µg/mL at 48 h the severity could significantly be reduced by half.

### 2.3. Seedlings under Semi-Controlled Conditions

**Figure 3:**
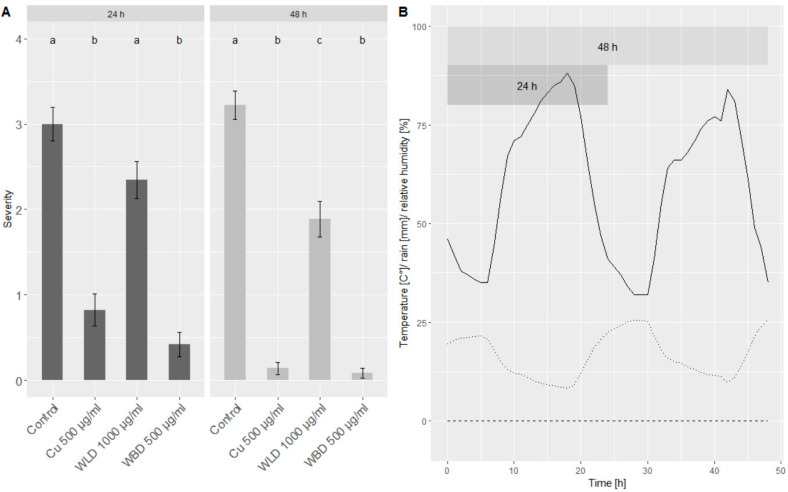
(**A**) Merged (cv. ‘Müller–Thurgau’ and ‘Pinot Noir’) results of the leaf disc assay conducted with seedling plants under semi-controlled conditions. Shown is the infection severity of leaf discs treated either with water (control), Cu-fungicide 500 µg/mL, WLD 1000 µg/mL or WBD 500 µg/mL: (0) no sporulation, (1) minimal sporulation, (2) low sporulation, (3) medium sporulation, (4) full sporulation. Welch’s anova and Games–Howell test for multiple comparison: Different letters indicate significant (*p* < 0.05; *n* = 72) differences between groups. (**B**) Weather conditions during the time of the experiment, when seedlings were placed in the field: Dashed line = rain, dotted line = temperature, solid line = relative humidity.

A preliminary test with seedling plants, cv. ‘Müller–Thurgau’ and ‘Pinot Noir’, was performed to study the protection efficacy of the WU extracts under field conditions. Seedlings were grown in greenhouse, treated in the lab, but then placed in the field in order to simulate a natural exposure to abiotic factors (e.g., sunlight, humidity/aridity). After 24 and 48 h, respectively, the seedlings were brought to the lab and a leaf disc assay was performed (Figure 3). While under field conditions, there was no rain, the mean temperature was 16.5 °C (max. = 25.6 °C, min = 8.3 °C) and the mean relative humidity was 59.0% (max. = 88%, min = 32%).

A mean severity of around three (medium sporulation) was observed for both time points (24 and 48 h) of the control treatment. Seedlings treated with 500 µg/mL of the Cu-fungicide showed a minimum sporulation (1) at 24 h and almost no sporulation (0) at 48 h. Compared to the control, when treated with 1000 µg/mL WLD, the DM severity could be slightly reduced: at 24 h by 22% and at 48 h by 40%. Using 500 µg/mL WBD, the severity, compared to the control treatment, could be reduced by 86% (24 h) and 98% (48 h), respectively, resulting in almost no sporulation.

### 2.4. Field Trials

**Figure 4:**
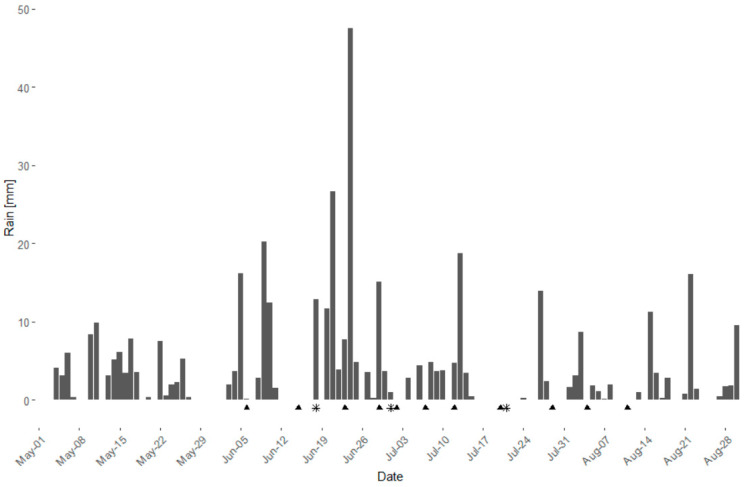
Daily rainfall (mm) during the season 2021 from May to August at the Julius Kühn-Institute facilities in Siebel-dingen, Rhineland-Palatinate, Germany. Triangles indicate date of plant protection application (∑ = 11). Asterisks indicate date of DM assessment (∑ = 3).

In the season 2021 a field trial in two vineyards (cv. ‘Riesling’ and ‘Dornfelder’) was conducted to test the efficacy of the WU extracts under field conditions. The season 2021 was characterized by multiple and partially heavy rainfalls (Figure 4); from May to August, 57 out of 123 days showed rainfall, with a total amount of 411.8 mm and a mean precipitation of 7.2 mm per rain day.

**Figure 5:**
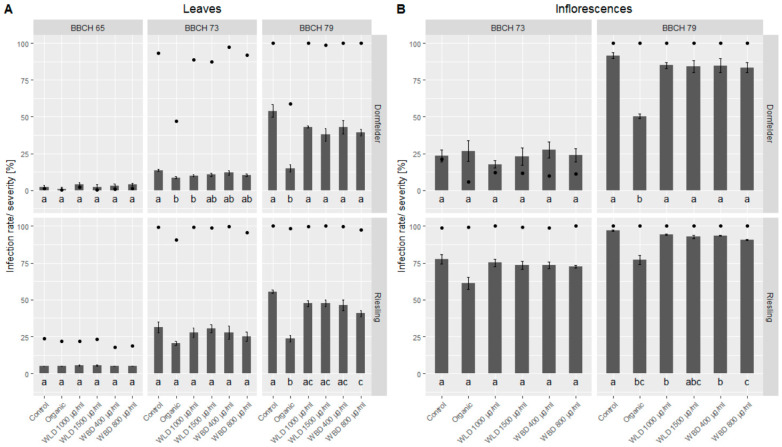
Mean DM infection rate (dots) and severity (bars) in the two trial fields, ‘Dornfelder’ and ‘Riesling’. (**A**) Leaves were assessed on three phenological stages (BBCH 65, BBCH 73, BBCH 79) and (**B**) inflorescences on two phenological stages (BBCH 73, BBCH 79). Welch’s anova and Games–Howell test for multiple comparison: Different letters indicate significant differences in mean infection severity; *p* < 0.05; ‘Dornfelder’: *n* = 5, ‘Riesling’: *n* = 4.

Due to massive and frequent rain events, DM primary infection and consequently multiple secondary infections occurred in the trial fields (Figure 5). In the untreated control blocks, the infection rate on leaves reached 100% in the last monitoring (at BBCH 79) with a severity of around 55%. This was true for both trial fields, ‘Dornfelder’ and ‘Riesling’. Also on inflorescences, the infection rate in both fields was 100% in the last assessment, with a severity of 91% and 97%, respectively.

Regarding leaf infection, on the first assessment (full flowering, BBCH 65), in both vineyards no differences in efficacy (based on infection severity) could be found between the different treatments (Table 1); however, DM infection severity (1.0–5.5%) in general was low at this time. Two weeks later, when berries had groat-size (BBCH 73) and the infection rate and severity increased, a slight protective effect of the organic treatment could be noticed with an efficacy of 36.3% for ‘Dornfelder’ and 33.3% for ‘Riesling’. Among the WU extracts, WLD 1000 µg/mL reached the highest efficacy at this stage with 26.6%. When majority of the berries were touching (BBCH 79), the efficacy of the organic treatment increased up to 71.7% in the ‘Dornfelder’ field and 57.2% in the ‘Riesling’ field. Here, the efficacy of the WU extracts varied between 13.8% (WLD 1500 µg/mL, ‘Riesling’) and 30.3% (WLD 1400 µg/mL, ‘Dornfelder’).

In the first assessment of the inflorescences (BBCH 73) the infection rate and severity differed between the two vineyards and so did the efficacy. No differences were found in the low infected ‘Dornfelder’ field between the five treatments, since infection severity was more or less the same for all treatments (17.8–27.4%). In the ‘Riesling’ field, the infection rate was almost around 100% in all treatments and according to the infection severity, the organic treatment revealed an efficacy of 20% while the WU extracts only showed an efficacy between 2.3% and 5.5% (Table 1). At BBCH 79 the infection rate of the inflorescences was 100% in all treatments of the ‘Dornfelder’ field. Here, the organic treatment had an efficacy of 45.2% and the WU extracts showed an efficacy between 7.0% and 9.0%. In the already highly infested ‘Riesling’ field, the infection severity of the inflorescences slightly increased, from BBCH 73 to BBCH 79, but the efficacy of the five treatments did not change over time; the organic treatment yielded 20.6% and the WU extracts reached 2.8% to 6.4%.

## 3. Discussion

Already decades ago, it has been shown that extracts made of *Warburgia* spp. are highly concentrated in compounds with antifungal activities [31,40]. The phytochemicals with the highest contribution to the antifungal properties of WU extracts belong to the group of sesquiterpenes; polygodial, warburganal and muzigadial [33,40]. In microtiter assays, their ability to inhibit the fungal growth could be demonstrated for *Candida utilis* (polygodial, MIC_50_ = 1.56 µg/mL; warburganal MIC_50_ = 3.13 µg/mL; muzigadial, MIC_50_ = 3.13 µg/mL) and *Saccharomyces cerevisiae* (polygodial, MIC_50_ = 0.78 µg/mL; warburganal MIC_50_ = 3.13 µg/mL; muzigadial, MIC_50_ = 1.56 µg/mL), among others [50]. Muzigadial could also inhibit growth of the filamentous fungi *Fusarium oxysporum* (MIC_100_ = 50 µg/mL) and *Aspergillus niger* (MIC_100_ = 5 µg/mL; 42). With *Warburgia salutaris* bark extract, Kuglerova et al. [41], in a microtiter assay, determined an MIC_50_ of 256 µg/mL for *Candida albicans*. The microtiter assay as performed in our study revealed an MIC_100_ (sporangia germination rate) for *P. viticola* of appr. 500 µg/mL for the WU leaf extract and appr. 5 µg/mL for the bark extract. For comparison, other plant extracts used against *P. viticola* expressed an MIC_100_ of 35 µg/mL (*Verbesina lanata*, 28), 24 µg/mL (*Juncus effusus*, 26) and 12 µg/mL (*Magnolia officinalis*, 29). The difference between the two MIC_100_ values by a factor of 100 indicates a higher concentration of antifungal active compounds in the bark of WU trees. With this in agreement, Abuto et al. [45], who tested WU leaf and bark extracts against *Staphylococcus aureus* and *C. albicans*, and in addition compared the phytochemical profile of the two plant parts, showed that bark extracts display a larger concentration of antimicrobial compounds than leaf extracts.

In the past, the antifungal properties of WU extracts were only proven against ascomycetes [31,33]. To our knowledge, our study is the first that the inhibitory activity of WU extracts could also be demonstrated against an oomycete. So far, inhibitory effects of crude WU extracts were only shown against fungi (*Candida albicans*, *Fusarium* spp., *Penicillium* spp.), bacteria (*Staphylococcus* spp., *Bacillus* spp., *Shigella boydii*, *Escherichia coli*, *Enterococcus faecalis*, *Mycobacterium* spp., *Neiserria gonorrhoea*) and *Plasmodium* spp. [34,41,43,44,51,52,53,54]. Our results together with the above studies indicate that WU extracts in fact exhibit a much broader antimicrobial activity and antifungal activity could also be expected against other fungal phytopathogens in viticulture, for example *Erysiphe necator* (Powdery Mildew) or *Botrytis cinerea* (Grey Mould).

Sprayed on grapevine leaf discs, WU leaf extracts suppressed DM infection at 500 µg/mL, but efficacy was closely linked to the time point of infection. No long-term protection was noted at this concentration. On the other hand, WU bark extracts could protect the leaf discs from immediate infection at 50 µg/mL. At 500 µg/mL, the bark extract could even suppress DM development for up to 48 h. The same effect could be seen under semi-controlled conditions, when seedling plants were exposed to field conditions for 24 and 48 h, respectively, after treatment. Here, WLD with 1000 µg/mL also expressed protective abilities against DM; efficacy, however, was limited. In a leaf disc bioassay, extracts based on *Pinus pinaster* knot at 500 µg/mL could fully suppress DM infection [55]. Using *Juncus effusus* medulla extract and *Magnolia officinalis* bark extracts, Thuering and colleagues [26,29] could reduce DM infection on grapevine seedlings with an efficacy of >90% (256 µg/mL) and 97% (1000 µg/mL), respectively. In general, extracts made of tree bark seem to be highly effective against DM. Mulholland et al. [56] tested bark extract from eight important northern forestry species on grapevine seedlings and all of them expressed significant inhibitory activity (between 50% and 98% efficacy) against DM at 1000 µg/mL. Furthermore, the authors identified several compounds with high efficacies against DM, namely larixyl acetate, larixol, lariciresinol, lariciresinol acetate and 15-hydroxydehydroabietic acid.

Although promising results were achieved in our laboratory and greenhouse tests, the efficacy of the WU extracts was distinctly reduced under field conditions. Among the tested conditions, the highest efficacy (30.3%) was reached in the ‘Dornfelder’ field at BBCH 79 by spraying leaves with WLD 1500 µg/mL. In contrast, in the ‘Riesling’ trial this treatment had the lowest efficacy (13.8%) among the WU extracts also at BBCH 79. For WBD 800 µg/mL, the leaf assessment resulted in similar efficacies for both trials, 25.2% (‘Dornfelder’) and 26.5% (‘Riesling’) at BBCH 79. For the inflorescences efficacy data of all tested WU extracts were beneath 10% at BBCH 79. Especially the low efficacies of the bark extracts at 800 µg/mL were unexpected, because in the preliminary tests 500 µg/mL WBD could effectively suppress DM infection on leaf discs and seedlings for up to 48 h. Basically, discrepancies in inhibition efficacy between controlled and field conditions are well known and might be due to several factors. Bark extract of *M. officinalis*, for instance, showed efficacy of 97% when used on grapevine seedlings under controlled conditions [29], while under field conditions the extract reached only 26% efficacy. Furthermore, Dagostin and colleagues [24] reported an efficacy between 79.9% and 95.8% for *S*. *officinalis* extracts when treating seedlings in the greenhouse. In field trials, however, the efficacy decreased to 17.5% in the first trial year and to 20.0% in the second year. One main reason for the efficacy decline under field conditions is probably rainfall, which washes off the extracts from the plant surface. In a simulated rain experiment (30 mm/h), the efficacy of a *S*. *officinalis* extract declined by 50% when precipitation was 50 mm [24]. When exposed to 10 mm, the efficacy already dropped from 80% to 50%. In the same experiment with a PPP based on Cu-hydroxide, the efficacy only marginally decreased from 80% to 70%, demonstrating a distinctly higher rain resistance. This would explain why the efficacy of the Cu-based organic treatment in our field trials was higher than for the WU extracts. One possible solution to improve rain resistance could be the use of proper formulations, like encapsulation [57,58]. Another option to improve the efficacy of the extracts in the field could be the addition of proper adjuvants, like silicon surfactants, that increase coverage and persistence on the plant [59,60,61]. Not only rain resistance, but also UV-stability of the WU extracts require further investigation to determine a suitable formulation that can improve the efficacy. Increasing the concentration of the antimicrobial compounds in the extract could be another way for a higher effectiveness. This could be reached either by choosing novel, innovative extraction methods or by certain cultivation practices that lead to higher synthesis of secondary metabolites in the leaves/bark [62,63,64].

In conclusion, under controlled and semi-controlled conditions low concentrations of leaves (1000 µg/mL) and bark (500 µ/mL) extracts of the African tree *W. ugandensis* could effectively prevent DM infection on leaf discs and seedlings, respectively. However, under field conditions the protective efficacy of the extracts against DM decreased significantly. Nevertheless, WU extracts may represent a suitable alternative to Cu-based fungicides in being nontoxic, sustainable and cost-effective. Future emphasis shall be on finding the particular formulations for the extracts in order to increase the rain resistance and thus the efficacy under true field conditions, including conditions of high infection pressure.

## 4. Material and Methods

### 4.1. Plant Material & Extraction

The plant material (leaves and barks) of *Warburgia ugandensis* was obtained from a medicinal plant farm in Pakwach district, Western Uganda. Exports of the plant material for research purposes was reported to and approved by the Ugandan authorities. For bark, 5–8 cm wide strips of bark were carefully peeled off the sapwood lengthwise. This procedure prevents the tree from dying. The leaves were harvested by pruning the WU shrubs, analogous to a hedge trimming. Both leaves and thin twigs were cut off. Then, both bark and leaves were washed and afterwards dried in the shade for about 7 days. When the moisture content was below 12%, the leaves were carefully stripped from the twigs and packed in air-permeable bags. They were stored in a dark room at a humidity below 15% to prevent rot formation. Five kg of fresh leaves yielded about 1 kg of dried leaves.

The plants for the WU plantation were obtained from a nursery of the state-owned National Forestry Resources Research Institute (NaFORRI) in Kifu (Uganda). The seeds were collected in the wild, exclusively by experts authorized for this purpose.

For the preparation of the extracts, both leaves and bark were ground to a particle size of 50 µm (IKA^®^ M20 Universal Mill, VWR International GmbH, Darmstadt, Germany). In each case, 100 g of the pulverized leaves or bark was mixed with 900 mL dichloromethane (Roth^®^, Karlsruhe, Germany). The extraction mixtures were incubated for 72 h with gentle shaking (100 rpm). Afterwards, the extraction mixtures were filtered and concentrated with a rotary evaporator (Rotavapor^®^ RE 111, Büchi Labortechnik GmbH, Essen, Germany) at a temperature of 50 °C until all dichloromethane was evaporated. Extract yields were 12–14% (*w*/*w*) for leaves and 8% (*w*/*w*) for barks, based on the raw plant material mass input.

### 4.2. Pathogen Cultivation

*Plasmopara viticola* was cultivated on grapevine (*Vitis vinifera*) seedlings cv. ‘Müller–Thurgau’ in a climate chamber. The climate conditions were 22 °C and 70% relative humidity with a 14/10 h day/night rhythm. Plants with 6–8 leaves were inoculated by spraying a sporangia suspension (20,000–30,000 sporangia/mL) on the abaxial site of the leaves. Subsequently, the whole plants were moistened with water, also by spraying, and packed in plastic bags. The plants were incubated for 24 h in the climate chamber before the plastic backs were removed. After, the plants were incubated 5–7 days in the climate chamber until oil spots were visible. Next, the adaxial site of the leaves were moistened with water and the plants were packed again in plastic bags. A total of 24 h later, the bags were carefully removed and sporangia were noticed on the abaxial site of the leaves. The cultivation process of *P. viticola* was repeated weekly to obtain fresh sporangia for the experiments.

### 4.3. In Vitro Bioassays

Sets of 96-well plates were used for the in vitro bioassays to determine the minimal inhibitory concentration 50 (MIC_50_) and 100 (MIC_100_) of the WU extracts against *P. viticola*. All extracts were dissolved in DMSO (Carl Roth GmbH, Karlsruhe, Germany) to achieve a stock concentration of 60 mg/mL. After this, a serial of nine dilutions were set up with H_2_O_dd_ to reach the following end concentrations in the wells: 0.05, 0.1, 0.5, 1, 5, 10, 50, 100, 500 µg/mL. From infected leaves, fresh sporangia were rinsed off with H_2_O_dd_ and the suspension was adjusted to 20,000–30,000 sporangia/mL. In each well, 100 µL of the diluted extract was mixed with 100 µL of the sporangia suspension and the 96-well plate was incubated at room temperature on the bench. As a negative control, H_2_O_dd_ with DMSO (same amount as for the extract dilutions) was used and a Cu-based fungicide (active agent: 537 g/kg Cu hydroxide = 350 g/kg Cu^2+^; Funguran^®^ progress, Biofa AG, Münsingen, Germany) as the comparative PPP. In one experiment, each treatment and concentration, respectively, had four replicates and the experiment was repeated four times.

The zoospore behavior and the sporangia germination rate were chosen to quantify the inhibitory effect of the tested products. The former was observed 1 h after incubation and rated according to three categories: (0) normal zoospore motion; (−1) no or unusual zoospore motion; (−2) no zoospore release. The sporangia germination rate was calculated 24 h after incubation by checking 50 sporangia per well for zoospore release.

### 4.4. In Vivo Bioassays

Leaf discs from greenhouse seedlings cv. ‘Müller–Thurgau’ and ‘Pinot noir’ were used for in vivo bioassays. Leaves were detached from the plants and washed with H_2_O_dd_. For each cultivar, the required amount of leaf discs for one experiment was cut from leaves with a cork borer (15 mm diameter), collected in a box and mixed. Per treatment variant, 12 random leaf discs, six from each cultivar, were put in a petri dish with wet filter paper, the abaxial site up.

**Figure 6:**
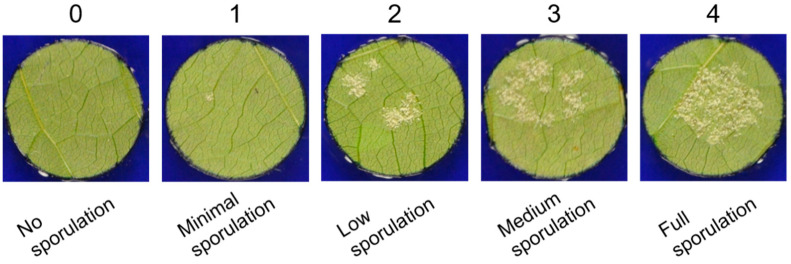
Severity categories used in this work to evaluate the degree of infection on grapevine leaf discs.

As descripted above for the in vitro assays, three dilutions for each extract were made: 5, 50, 500 µg/mL. The extracts were sprayed horizontally on the leaf discs by using an application chamber (SprayLab Epilogic, Schachtner Gerätetechnik, Ludwigsburg, Germany) with following settings: 32 cm distance from leaf discs to nozzle, 3.0 bar pressure, 2.5 km/h spraying device pace, 300 L/ha application rate. Control leaf discs were sprayed with H_2_O_dd_ containing DMSO (the same amount as for the extract dilutions) and for comparison, a Cu-based fungicide (Funguran^®^ progress, Biofa GmbH, Münsigen, Germany) with the same concentrations as the extracts was applied. After application of each treatment, the spraying device was cleaned with H_2_O_dd_. The sprayed leaf discs were arranged on a 0.8% H_2_O_dd_-agar plate (Agar-Agar, Carl Roth, Karlsruhe, Germany) with abaxial site up and inoculated with a 70 µL drop of a sporangia suspension (20,000–30,000 sporangia/mL). Subsequently, the agar plates with the inoculated leaf discs were incubated in the climate chamber at the same conditions as for the pathogen cultivation. A total of 24 h after inoculation, the drops were removed from the leaf discs with a vacuum device (Mini-Vac, Axon Lab AG, Stuttgart, Germany). Then, plates were put back to the climate chamber for further incubation. The full sporangiophore formation was noticed about 10 days after inoculation. At this time, the infection severity on the leaf discs was evaluated based on five severity categories: (0) no sporulation; (1) minimal sporulation; (2) low sporulation; (3) medium sporulation (4) full sporulation (Figure 6). In order to investigate the temporal protective effect of the extracts, the sprayed leaf discs were inoculated with sporangia either directly after the treatment (0 h), 24 h after the treatment or 48 h after the treatment. For each time point, the experiment was repeated four times. Statistical analysis of the data was done with RStudio [65]. With Welch’s anova and Games–Howell test for multiple comparison, a non-parametric approach was chosen for statistical analysis since the obtained data (infection severity) were not normally distributed and showed heteroscedasticity.

### 4.5. Tests under Semi-Controlled Conditions

Greenhouse seedlings cv. ‘Müller–Thurgau’ and ‘Pinot noir’ were used to test the efficacy of the extracts under field conditions. The whole plants (10 to 15 leaves) were treated in the SprayLab application chamber using a tunnel spraying device with one nozzle on each side and following settings: 55 cm distance from nozzle to nozzle, 5.0 bar pressure, 1.0 km/h spraying device pace, 400 L/ha application rate. The treatments were as follows: Control (H_2_O_dd_ with DMSO), Cu-fungicide (Funguran^®^ progress, Biofa GmbH, Münsigen, Germany) 500 µg/mL, WBD 500 µg/mL, WLD 1000 µg/mL. For each treatment, six seedlings cv. ‘Müller–Thurgau’ and six seedlings cv. ‘Pinot noir’ were taken. After the treatment, seedlings were put outside in front of the greenhouse. A total of 24 h later, half of the seedlings were brought to the lab for inoculation with *P. viticola*, the other half after 48 h. From each plant, four leaves were randomly removed and from each leaf three leaf discs were cut. In total, 72 leaf discs per treatment and time point (24 and 48 h) were prepared, inoculated and evaluated as described in the Section 4.4 *In Vivo Bioassays*. Here as well, Welch’s anova and Games–Howell test for multiple comparison were chosen for statistical analysis.

### 4.6. Field Trials

Two vineyards were selected for the field trials in 2021. Both were in close vicinity to the Julius Kühn-Institute facilities in Siebeldingen, Rhineland-Palatinate, Germany. One vineyard was planted with the white grapevine cultivar ‘Riesling’ (49°13′44.6″ N 8°00′25.6″ E). The second vineyard was planted with the red cultivar ‘Dornfelder’ (49°12′55.1″ N 8°02′08.9″ E). Both vineyards were established on loamy soil and the plants were trained in vertical shoot positioning.

A randomized block design was chosen for the trial and the experiment was conducted based on the EPPO guidelines for *P. viticola* (PP 1/31(3)). The ‘Riesling’ vineyard consisted of 24 rows with 25 plants per row. For each treatment, four rows were randomly picked resulting in 100 plants per PPP. The ‘Dornfelder’ vineyard consisted of 10 rows with 60 plants per row. Here, each of the rows were divided into three blocks and for each treatment, five blocks were randomly picked resulting also in 100 plants per PPP.

Six treatments were set up for the field trial: Untreated control, organic treatment, WBD 400 µg/mL, WBD 800 µg/mL, WLD 1000 µg/mL, WLD 1500 µg/mL. The vines in the untreated control received no protection spraying. For the organic treatment, a mixture of Cu-based PPP (2 g/L, Funguran^®^ progress) against DM plus net sulfur (9 g/L, Stulln, Biofa AG, Münsingen, Germany) against Powdery Mildew (*Erysiphe necator*) was used. The bark and leaf extracts were dissolved in DMSO to achieve a stock concentration of 250 mg/mL and with tap water the final spraying concentrations were prepared.

During the season, eleven plant protection applications took place using a spraying device for tractors (parcel sprayer, Schachtner Gerätetechnik, Ludwigsburg, Germany) carrying six pressure tanks with 25 L volume (Figure 2). Spraying took place in a tunnel with five nozzles on each side. While spraying, the tractor had a constant speed of 4 km/h resulting in a spraying amount of about 400 L/ha.

DM assessments were made on three dates during the season 2021: 18 June (BBCH 65, full flowering), 1 July (BBCH 73, berries groat-sized) and 21 July (BBCH 79, majority of berries touching). On the first date, only leaves were assessed, and on the second and third, leaves and inflorescences. In each treatment block, 100 randomly picked leaves/inflorescences were evaluated for DM infection. The infection rate (percentage of infected leaves/inflorescences) and severity (percentage of infected leaf/inflorescences area) was determined. Furthermore, the efficacies of the treatments were calculated according to Abbott [66]: [1 − (A × B^−1^)] × 100; with ‘A’ as the infection severity of the treatment block and ‘B’ as the infection severity of the control block.

Statistical analysis was done using the infection severity data and the program R. The grouped data set (by means of assessed plant part, cultivar, assessment date) were not normally distributed and mainly showed homoscedasticity. However, since the sample size was low (‘Dornfelder’: *n* = 5; ‘Riesling’: *n* = 4) Welch’s anova and Games–Howell test for multiple comparison were chosen to find significant differences between the treatment groups.

### 4.7. Weather Data

Because climatic conditions, especially rainfall, is an important driving force in the epidemiology of *P. viticola,* we collected data from a weather station close to the Julius Kühn-Institute facilities (49°12′59.4″ N 8°02′52.6″ E). The station is run by the DLR Rhineland-Palatinate (www.dlr.rlp.de, accessed on 1 December 2021).

## Figures and Tables

**Table 1:** Efficacy (%) of the WU extracts based on the assessment data (only infection severity) from the trial field experiment conducted in 2021 in two vineyards (‘Dornfelder’ and ‘Riesling’). Shown is the mean efficacy (±sd) on three and two, respectively, different phenological stages of each tested treatment: Organic, WLD 1000 µg/mL, WLD 1500 µg/mL, WBD 400 µg/mL and WBD 800 µg/mL.

				Leaf		Inflorescences	
		*n*	BBCH 65	BBCH73	BBCH 79	BBCH 73	BBCH 79
Organic	‘Dornfelder’	5	40.0 ± 54.8	36.3 ± 12.5	71.7 ± 11.4	−33.7 ± 102.5	45.2 ± 2.2
	‘Riesling’	4	0.0 ± 0.0	33.3 ± 10.1	57.2 ± 8.4	20.0 ± 17.4	20.6 ± 6.5
WLD 1000 µg/mL	‘Dornfelder’	5	16.0 ± 47.7	26.6 ± 4.3	18.0 ± 16.8	18.1 ± 34.0	7.0 ± 7.8
	‘Riesling’	4	−10.0 ± 11.5	6.8 ± 34.2	14.2 ± 9.4	2.3 ± 11.9	2.8 ± 0.5
WLD 1500 µg/mL	‘Dornfelder’	5	40.0 ± 54.8	21.9 ± 14.9	30.3 ± 14.4	−3.4 ± 65.2	8.4 ± 8.1
	‘Riesling’	4	−10.0 ± 11.5	−1.0 ± 28.0	13.8 ± 9.4	4.6 ± 11.6	4.4 ± 2.0
WBD 400 µg/mL	‘Dornfelder’	5	3.3 ± 7.5	9.6 ± 33.9	19.1 ± 22.1	−23.8 ± 66.4	7.6 ± 7.7
	‘Riesling’	4	0.0 ± 0.0	8.2 ± 35.9	16.7 ± 13.1	4.2 ± 14.6	3.6 ± 1.0
WBD 800 µg/mL	‘Dornfelder’	5	3.3 ± 7.5	21.9 ± 20.9	25.2± 18.3	−3.1 ± 29.1	9.0 ± 5.2
	‘Riesling’	4	0.0 ± 0.0	17.9 ± 28.0	26.5 ± 7.0	5.5 ± 10.1	6.4 ± 0.5

## Data Availability

Data collected in this study are available from the corresponding author upon request.
